# Host plant-driven sensory specialization in *Drosophila erecta*

**DOI:** 10.1098/rspb.2013.0626

**Published:** 2013-06-07

**Authors:** Jeanine Linz, Amelie Baschwitz, Antonia Strutz, Hany K. M. Dweck, Silke Sachse, Bill S. Hansson, Marcus C. Stensmyr

**Affiliations:** Department of Evolutionary Neuroethology, Max Planck Institute for Chemical Ecology, Hans-Knöll-Strasse 8, 07745 Jena, Germany

**Keywords:** insect olfaction, specialization, *Drosophila*, oviposition

## Abstract

Finding appropriate feeding and breeding sites is crucial for all insects. To fulfil this vital task, many insects rely on their sense of smell. Alterations in the habitat—or in lifestyle—should accordingly also be reflected in the olfactory system. Solid functional evidence for direct adaptations in the olfactory system is however scarce. We have, therefore, examined the sense of smell of *Drosophila erecta*, a close relative of *Drosophila melanogaster* and specialist on screw pine fruits (*Pandanus* spp.). In comparison with three sympatric sibling species, *D. erecta* shows specific alterations in its olfactory system towards detection and processing of a characteristic *Pandanus* volatile (3-methyl-2-butenyl acetate, 3M2BA). We show that *D. erecta* is more sensitive towards this substance, and that the increased sensitivity derives from a numerical increase of one olfactory sensory neuron (OSN) class. We also show that axons from these OSNs form a complex of enlarged glomeruli in the antennal lobe, the first olfactory brain centre, of *D. erecta*. Finally, we show that 3M2BA induces oviposition in *D. erecta*, but not in *D. melanogaster*. The presumed adaptations observed here follow to a remarkable degree those found in *Drosophila sechellia*, a specialist upon noni fruit, and suggest a general principle for how specialization affects the sense of smell.

## 1. Introduction

Because the sense of smell directly interfaces with the environment, it is an ideal system to study adaptive responses to altered environmental conditions and shifts in habitat preference. An animal exposed to a novel environment or niche will presumably alter its olfactory system over evolutionary time to encompass the composition of new chemical volatiles. Insects are well suited for this line of study, because they possess a rich repertoire of odour-guided behaviours (such as mating and breeding), and their nervous system is accessible as well as numerically reduced compared with the nervous system of vertebrates [1].

The Seychelles endemic *Drosophila sechellia* (*melanogaster* species subgroup, subgenus *Sophophora*)—a close relative of the laboratory work-horse *D. melanogaster*—is a well-known model system for studying questions relating to adaptive host specialization, particularly regarding the olfactory system [2–8]. The *D. sechellia*–*Morinda* system has, however, a number of shortcomings as a model of host plant-driven sensory specialization. Ample evidence suggests that *D. sechellia* has a very small effective population size [9–11], accordingly, observed changes to its chemosensory makeup may be the results of random processes (such as genetic drift) rather than adaptations. In addition, there are also valid concerns over the antiquity of the *Morinda* association ([12]; but see [13]). In order to shed further light on the mechanisms underlying the evolution and adaptation of olfactory systems and to pinpoint processes involved in specialization, we have here investigated the *Drosophila erecta*–*Pandanus* association, the second specialized insect–host system of the *melanogaster* species subgroup.

**Figure 1.**
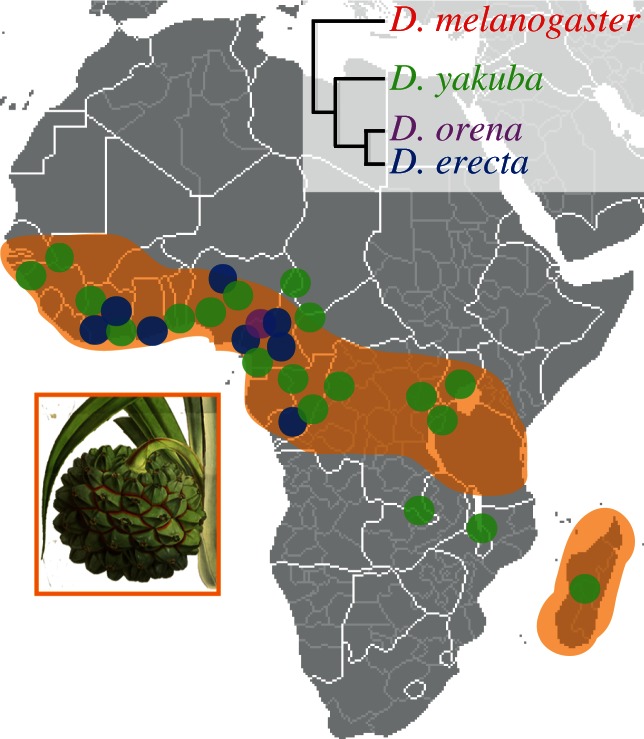
Geographical distribution of *D. erecta*, its sympatric siblings and the genus *Pandanus* in tropical Africa. The four *melanogaster* sibling species differ in their ecology, distribution and phylogenetic relationship. *Drosophila erecta* occurs in swampy and coastal habitats of western-central Africa, and uses fresh *Pandanus* spp. fruits as a food source and breeding site. Its closest relative, *Drosophila orena* (known from a single collection event on Mount Lefo, Cameroon), has an unknown ecology. *Drosophila melanogaster* (cosmopolitan) and *D. yakuba* (endemic to tropical Africa) are generalists. Map by courtesy of Wikipedia; modified from ([15,16]; http://www.mobot.org). *Pandanus candelabrum* image adapted from an original illustration. Reproduced with the kind permission of the Director and the Board of Trustees, Royal Botanic Gardens, Kew.

*Drosophila erecta* is endemic to gallery forests of west-central Africa (Ivory Coast, Nigeria, Cameroon and Congo) and specializes on ripe fruits of *Pandanus* spp. Parkinson (Pandanaceae; [14,15]; figure 1). The oft-mentioned specialization of *D. erecta* towards a single host—*Pandanus candelabrum*—is not correct. The confusion stems from the fact that many *Pandanus* species are taxonomically very hard to distinguish [17]. From the 20 to 24 described *Pandanus* species that occur in continental tropical Africa [15,17], *D. erecta* uses at least three, and is presumably able to use fruits from all *Pandanus* species that bear fleshy syncarps [18–20]. The geographical distribution of *D. erecta* also largely overlaps with the occurrence of the genus *Pandanus* in Africa (figure 1). *Pandanus* trees fruit only once a year over a period of about two months, an attribute that has resulted in *D. erecta* being termed a seasonal specialist [15]. When *Pandanus* fruits are available, *D. erecta* can be viewed as a specialist; however, in times of *Pandanus* shortage, *D. erecta* switches to other food sources and breeding sites. During a 40-month study, Lachaise & Tsacas [14] found a small number of *D. erecta* on fungi, *Ficus capensis* fruits and, once, on a palm bud. In another study [15], a handful of *D. erecta* (but many *D. melanogaster* and *Drosophila yakuba*) were trapped in banana baits between two *Pandanus* fruiting events.

In contrast to *D. erecta*, its sibling *D. sechellia* relies exclusively on fruits of the noni tree (*Morinda citrifolia*, Rubiaceae) for feeding and breeding [19,21]. Fresh noni fruits are hostile to most drosophilids, including its generalist cosmopolitan relatives *D. melanogaster* and *Drosophila simulans. Pandanus* syncarps are also visited by other drosophilids, including the sympatric sibling species *D. yakuba* (figure 1), a generalist that prefers *F. capensis* (Moraceae), *Theobroma cacao* (Malvaceae) and *Landolphia hirsuta* (Apocynaceae) [22]. Although, *D. yakuba* (like most of the other visiting drosophilids) uses *Pandanus* fruits only for feeding and not for breeding [14]. By contrast, nothing is known about the ecology of its closest relative, *Drosophila orena*, which is only known from a single collection event on Mount Lefo, Cameroon.

Here, we ask whether the specialized lifestyle of *D. erecta* is reflected in its olfactory system. We demonstrate that the *D. erecta*–*Pandanus* association has resulted in alterations in the olfactory system, similar to those found in *D. sechellia*. Specifically, we show that *D. erecta*, in contrast to its non-specialized sympatric relatives *D. orena*, *D. yakuba* and *D. melanogaster*, is more sensitive towards the *Pandanus* volatile 3-methyl-2-butenyl acetate (3M2BA; also known as prenyl acetate). The enhanced sensitivity is accomplished by increasing a specific class of olfactory sensory neurons (OSNs); intriguingly, this is the same class of OSNs affected in *D. sechellia*. Moreover, we show that the numerical increase in the periphery is accompanied by the formation of enlarged glomeruli in the antennal lobes (ALs), the first olfactory neuropil in the fly brain. Furthermore, 3M2BA triggers oviposition in *D. erecta*.

## 2. Material and methods

### (a) Flies

Wild-type flies were obtained from the *Drosophila* Species Stock Centre (https://stockcenter.ucsd.edu): *D. erecta* (14021–0224.01), *D. orena* (14021–0245.01) and *D. yakuba* (14021–0261.01); except for *D. melanogaster* (wild-type Berlin). For functional imaging, transgenic fly lines of *D. melanogaster* used were Orco-Gal4 [23] and UAS-G-CaMP3.0 [24]. *GCaMP3.0* flies were kept for 36 h at 29°C before experiments to enhance UAS-reporter gene expression. All flies were reared on standard cornmeal medium at 25°C, except for *D. orena* (18°C), 12 L : 12 D photoperiod and 70 per cent relative humidity. All experiments were carried out with mated female flies, 4–10 days post-eclosion, except for oviposition experiments.

### (b) Fruit headspace collections

Ripe *Pandanus* sp. fruits were wrapped in polyester bags (Toppits Bratschlauch, Germany), and volatiles were trapped with Super Q adsorbent filters (30 mg; Alltech, Deerfield, IL, USA). Sample collection was done for 6 h (1.0 l min^−1^) using a vacuum pump (Apex Pro, Casella, UK). Adsorbed volatiles were desorbed by eluting filters with 300 µl dichloromethane (DCM, 99%; Roth). Samples were stored at −20°C.

### (c) Chemical analysis

*Pandanus* fruit volatiles were analysed by gas chromatography-mass spectrometry (GC-MS) (Agilent 6890 GC, and 5975 MS). The GC was equipped with a non-polar HP5 column (30 m × 0.25 mm ID, 0.32 µm film thickness; Agilent) with helium as a carrier gas (1.1 ml min^−1^ flow rate). One microlitre of sample was injected splitless at 265°C. Temperature program was 40°C for 3 min, rising to 280°C at 5°C min^−1^, held for 10 min. Compounds were identified by their mass spectra in a National Institute of Standards and Technology library search, and were confirmed by comparing the Kovats index with the indices of synthetic compounds.

### (d) Electrophysiology

Gas chromatography coupled with electroantennographic detection (GC-EAD) and electroantennography (EAG) [25] were used to identify the antennal responses of the four sibling species to the collected *Pandanus* fruit volatiles. Flies were mounted following standard procedures [26]. For GC-EAD experiments, 1 µl of *Pandanus* extract or synthetic compound (*ca* 100 ng µl^−1^, in DCM), was injected splitless into an Agilent GC 6890 equipped with a non-polar column (for details see above). A 1 : 1 effluent splitter allowed for the simultaneous flame ionization detection (FID) and the EAD of the separated compounds. Helium was the carrier gas; injector and detector temperatures were 250°C and 300°C, respectively. Column temperature was held at 40°C for 1 min, rising to 300°C at 20°C min^−1^, held for 10 min. The GC-separated components were introduced into a continuous, filtered and humidified air stream flowing over the antennae (1 l min^−1^). The EAD and FID signals were simultaneously recorded and analysed (GcEad-1.2.0, Syntech, Hilversum, The Netherlands).

Determination of GC-EAD active compounds was simplified by converting the antennal responses into false-colour-coded heat maps using Fiji [27]. Therefore, EAD traces (exported in ASCII code) of single flies were imported into Fiji (as ‘text image’) and colour-coded (‘smart’). Headspace compounds were determined as biologically active when eliciting reproducible responses in the fly antennae. EAD responses were manually quantified with the Syntech software [28], and normalized to the first external solvent-elicited peak.

Principal component analysis (PCA) was then applied (variance–covariance matrix), and the antennal responses were displayed within a three-dimensional space. With one-way analysis of similarity (ANOSIM), we tested by which degree the antennal responses of the four species differed from each other (Bray–Curtis similarity, sequential Bonferroni correction, 10 000 permutations). The similarity percentage (SIMPER) method [29] indicates compounds contributing to this dissimilarity [26]. Statistics were carried out with the software package PAST v. 2.11 [30].

For EAG experiments, synthetic compounds were diluted in mineral oil (Sigma-Aldrich, Steinheim, Germany) in decadic steps (10^−1^ to 10^−5^) to record dose–response curves. *Prior to* each experiment, 10 µl of diluted odours was freshly loaded onto a small piece of filter paper (1 cm^2^, Whatman, Dassel, Germany), and placed inside a glass Pasteur pipette. The antennae were held in a continuous, filtered and humidified air stream produced by a stimulus controller (Stimulus Controller CS-55, Syntech), whose flow was 1 l min^−1^. Odour stimuli were applied for 0.5 s into the constant air stream. Antennal signals were recorded with EagProV2 (Syntech). Control stimuli consisted of filter paper with mineral oil, which was applied before each odours set. Response traces were baseline corrected with the mean of the first second and normalized to the control. Statistics (paired Student's *t*-test; one-way ANOVA with Turkey's post hoc test) were done with the software InStat v. 3.06 (GraphPad Software, Inc., San Diego, CA, USA).

### (e) Functional imaging

To specify which glomerulus is activated by 3M2BA, we performed functional calcium imaging experiments as previously described [31]. Pure odorants were diluted (10^−5^, 10^−3^, 10^−1^) in mineral oil (BioUltra, Sigma-Aldrich).

### (f) Single sensillum recordings

To test the activation of the Or22a receptor by different 3M2BA concentrations, dose–response experiments were performed on the large basiconic sensilla in *D. erecta* and *D. melanogaster* females. Pure odorants were diluted (10^−2^–10^−8^) in mineral oil (BioUltra, Sigma-Aldrich). Single sensillum recording (SSR) measurements were performed as outlined in [32]. Responses to the solvent control were subtracted.

### (g) Three-dimensional reconstruction of the antennal lobe

Anesthetized flies were dissected in *Drosophila* ringer solution as described by Wu & Luo [33]. The brains were fixed in 4 per cent PFA (4% paraformaldehyde, 0.1 M phosphate buffer, 0.2% Triton X-100) for 30 min on ice, and washed 3 × 20 min in PBST at room temperature (RT). Pre-incubation in PBST-NGS (PBST + 5% normal goat serum) lasted 1 h at RT. Brains were incubated in 1 : 30 mouse monoclonal nc82 antibody (the Developmental Studies Hybridoma Bank) in PBST-NGS, for 2 days at 4°C. After being washed 3 × 20 min at RT with PBST, brains were incubated in the secondary antibody, 1 : 200 goat anti-mouse Alexa Fluor 633 (Invitrogen, Darmstadt, Germany) in PBST-NGS, for 2 days at 4°C. Afterwards, brains were washed 3 × 20 min at RT with PBST, and mounted in Vectashield (Vector Laboratories, Burlingame, CA, USA) on glass slides using spacers made of cover slides.

Whole-brain mounts were studied with a Zeiss LSM 510 Meta confocal laser scanning microscope (Carl Zeiss, Jena, Germany). Scans were performed for every 0.5 µm stack with a 40 × 10 water immersions objective (C-Apochromat 40x/1.2 W UV-Vis-NIR; Carl Zeiss). Structures labelled with Alexa Fluor 633 were excited with a HeNe laser at 633 nm.

Three-dimensional reconstructions and volumetric measurements of glomeruli were made with the segmentation software AMIRA v. 5.4.1 (Visage Imaging GmbH, Berlin, Germany). The ALs of three *D. melanogaster* and *D. erecta* females were analysed and compared. For each species, a template AL was chosen based on staining quality and shape. The terminology of the glomeruli is based on Couto [34]. Per species, the glomeruli volume was normalized to the total volume of the AL, which was equal in both species (*D. melanogaster* 63638.14 ± 3992.36 s.e.; *D. erecta* 68111.61 ± 3847.56 s.e.).

### (h) Neuronal backfill

To trail the convergence of the OSNs of the ab3 sensilla, anterograde-neurobiotin backfills (Molecular Probes, Carlsbad, CA, USA) of *D. erecta* females were performed as previously described [5]. Treated flies were prepared for whole-brain mount confocal microscopy, as explained above (see §2*g*).

### (i) Oviposition site preference

By using a two-choice assay, we tested the oviposition site preference (OSP) of the generalist *D. melanogaster* and the specialist *D. erecta* to 3M2BA. Tests were generally performed as previously described [32] with some modifications: indication was given that additional spatial information influences egg-laying behaviour in the flies. We, therefore, created homogeneous vertical structures by (i) gently separating the medium from the plate edges with help of a scalpel, and (ii) pressing the odour containing cup inside the medium. Experiments were conducted at 25°C, 12 L : 12 D photoperiod, 70 per cent relative humidity. Per species, we used six cages of 30 flies. Flies were allowed to oviposit for 40 h. For analyses, eggs were counted at three different positions (vertical medium edge, horizontal medium surface and vertical gap at the odour cup).

To test for the effect of odour and spatial information on the OSP, linear mixed effects models were performed using the computing environment R v. 2.15.2 [35]. Spatial and odour information were determined as fixed, cage identity and treatment served as random factors, with treatment nested in cage identity. The full model (with interaction between odour and position) was simplified by removing fixed factors step by step. Significance values for the fixed factors were obtained by comparing the models with a likelihood ratio test. *Prior to* the analyses numbers of eggs were log-transformed.

## 3. Results and Discussion

### (a) Species-specific antennal detection of *Pandanus* volatiles does not reflect phylogeny

**Figure 2.**
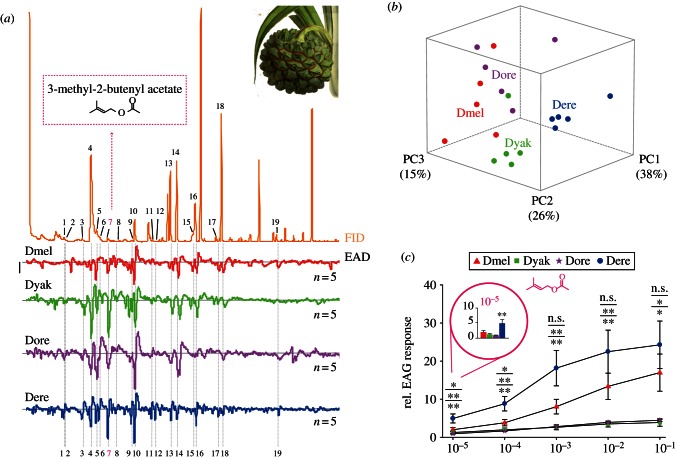
Antennal response spectra of the four *melanogaster* sibling species—evoked by the *Pandanus* sp. fruit headspace volatiles. (*a*) Headspace odour of *Pandanus* sp. fruits (upper part) and EAD responses (lower part). Bar stands for 1 mV of EAD response. Active compounds are coded as follows: 2,3-butanediol (no. 1), ethyl butyrate (no. 2), ethyl isovalerate (no. 3), isoamyl acetate (no. 4), 2-heptanone (no. 5), styrene (no. 6), 3M2BA (no. 7), ethyl tiglate (no. 8), 6-methyl-5-heptene-2one (no. 9), ethyl hexanoate (no. 10), linalool (no. 13), phenethyl alcohol (no. 14), benzyl acetate (no. 15), ethyl benzoate (no. 16), isopentyl hexanoate (no. 17), β-phenethyl acetate (no. 18). Peaks (no. 11), (no. 12) and (no. 19) are unidentified. Emphasized in pink is the novel *Drosophila* ligand 3M2BA (no. 7). (*b*) PCA (variance–covariance matrix) of quantified GC-EAD responses. PC1, 2 and 3 are plotted in three-dimensional space (79% cumulative variance). Species differ significantly from each other (one-way ANOSIM; Bray–Curtis distance; *R* = 0.78; *p* < 0.001). Isoamyl acetate (no. 4), 3M2BA (no. 7) and phenethyl alcohol (no. 14) most supported to the observed dissimilarity (SIMPER; all groups pooled; cumulative contribution 30.8%). (*c*) EAG dose–response curves of 3M2BA from the four species. *Drosophila erecta* is highly sensitive to 3M2BA, starting at 10^−5^ (paired *t*-test, **p < 0.01, emphasized). *Drosophila erecta* differs from its siblings (one-way ANOVA; Turkey's post hoc test; *p* > 0.05 n.s.; *p < 0.05; **p < 0.01). Mean ± s.e. In all graphs, species abbreviation and colour-code is as follows: *D. melanogaster* (Dmel, red), *D. yakuba* (Dyak, green), *D. orena* (Dore, violet) and *D. erecta* (Dere, blue), and *n* = 5 per species. *Pandanus candelabrum* image adapted from an original illustration. Reproduced with the kind permission of the Director and the Board of Trustees, Royal Botanic Gardens, Kew.

The purpose of this study was to unravel insect–host-derived adaptations within the olfactory system of *D. erecta* to its host, fresh fruits of the screw pine tree *Pandanus*. We, therefore, followed a comparative approach using electrophysiology in four sympatric *melanogaster* sibling species (*D. melanogaster*, *D. yakuba*, *D. erecta* and *D. orena*; figure 1) with different host specificity and ecology (in the case of *D. orena*, with unknown ecology). First, we sampled volatiles from fresh *Pandanus* sp. fruits. The collected headspace was then examined for antennal activity in linked GC-EAD experiments [25]. Performing GC-EAD experiments with *D. melanogaster*, *D. yakuba*, *D. orena* and *D. erecta* as natural odorant detectors, we found a total of 19 volatiles eliciting reproducible antennal responses among the *Pandanus*-emitted volatiles (see figure 2*a* and electronic supplementary material, figure S1). We identified the biologically active compounds via GC-MS, through library comparison and co-injection of synthetic standards. We succeeded in identifying all but three minor peaks (nos 11, 12 and 19).

Notably, the *Pandanus* bouquet evoked species-specific response spectra on the fly antenna. However, only few and typically fruit-related compounds [36] induced very strong responses in all species (see figure 2*a* and electronic supplementary material, figure S1). We next asked whether the antennal response spectra towards the *Pandanus* bouquet varied between the four different species. First, we examined if the antennal response spectra simply mirrored the phylogenetic relationship between these species. To address this question, we performed a PCA based on the quantitative EAD responses to the 19 active peaks in the *Pandanus* headspace, and plotted the results within three-dimensional space (figure 2*b*). The *D. erecta* measurements formed a distinct cluster, which did not overlap with *D. orena*, its closest relative. Instead, *D. erecta* grouped closer with *D. yakuba*, a species also attracted to *Pandanus* syncarps [22]. On the other hand, *D. orena* clustered closer to the generalist *D. melanogaster*. The PCA, hence indicates that the generalist *D. yakuba* and the specialist *D. erecta* show similarities in their antennal response spectra, perhaps owing to the common food source [14]. The distinct clustering of the four species was mainly based on differential EAG responses to the three compounds, isoamyl acetate (no. 4), 3M2BA (no. 7) and phenethyl alcohol (no. 14; figure 2*a*). We thus conclude that the recorded olfactory responses of the different fly species probably reflect lifestyle rather than phylogenetic relationship.

### (b) *Drosophila erecta* displays increased antennal sensitivity towards a key *Pandanus* volatile

Whereas, isoamyl acetate and phenethyl alcohol are common natural fruit ligands [36], 3M2BA attracted our particular interest. 3M2BA is fairly rare in nature, but has previously been reported as a diagnostic volatile from *Pandanus* syncarps [37]. Although 3M2BA was a relatively small constituent of the *Pandanus* fruit examined here, it evoked strong antennal responses in the GC-EAD experiments (figure 2*a*). Accordingly, we next examined the sensitivity of the four species towards this novel *Drosophila* odour ligand in more detail. In dose–response experiments, we recorded electroantennogram (EAG) activity from the four species, using synthetic 3M2BA as stimulus. The specialist *D. erecta* was considerably more sensitive than its relatives towards this *Pandanus* volatile (figure 2*c*). Our findings thus parallel earlier studies from other insects demonstrating increased sensitivity of specialist species towards host-specific volatiles [38,39]. In short, we assume that the increased sensitivity of *D. erecta* to 3M2BA represents a host-specific adaptation towards use of *Pandanus* syncarps. Next, we wondered which olfactory receptors (ORs) are activated by this ligand and where the respective OSNs target in the brain.

### (c) 3-methyl-2-butenyl acetate predominantly activates ab3A type olfactory sensory neurons

**Figure 3.**
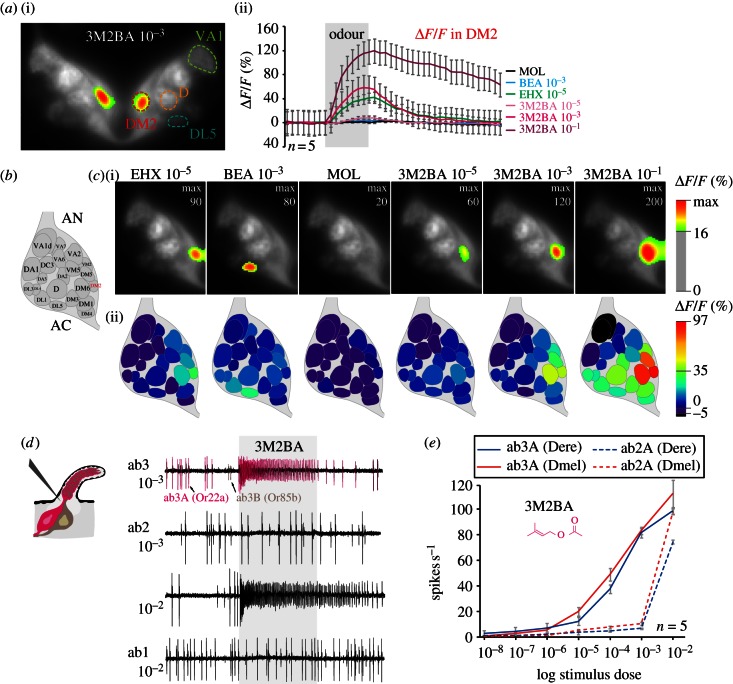
Identification of ORs activated by 3-methyl-2-butenyl acetate via functional imaging and single sensillum recordings. (*a*) Functional imaging in *D. melanogaster*. Representative recording of both ALs performed with 3M2BA (i), and activity traces of the DM2 glomerulus (ii) in response to the same set of odours as used in (*c*). Error bars represent s.d. (*b*) Glomerular atlas of the AL. (*c*) Representative recordings performed with different reference stimuli (EHX, ethyl hexanoate; BEA, benzaldehyde; and MOL, mineral oil) and different 3M2BA concentrations illustrate the strong activation of DM2 glomerulus by 3M2BA (i). Images are individually scaled to the strongest activated glomeruli. Values below the *ΔF*/*F* threshold are omitted to illustrate the specificity of the signals, as well as the glomerular arrangement as visualized by the intrinsic fluorescence. Corresponding odour-induced activity (average % *ΔF*/*F*) plotted on schematic ALs (ii). (*d*) Single sensillum recording (SSR) measurements of the large basiconic sensilla in female *D. erecta* illustrate strong activation of the Or22a receptor (ab3A neuron) by 3M2BA. At higher concentrations, Or59b (ab2A neuron) is also activated. (*e*) SSR dose–response experiments performed on ab2 and ab3 sensilla in *D. erecta* (blue: solid line, ab3A Dere; dashed line, ab2A Dere) and *D. melanogaster* (red: solid line, ab3A Dmel; dashed line, ab2A Dmel) females with 3M2BA. Mean ± s.e.

To identify OR(s) activated by 3M2BA, we next performed functional imaging of the AL. Briefly, the AL consists of subunits, the so-called glomeruli, which are formed by afferents of the OSNs; these in turn are housed in the sensilla that cover the insect antenna. OSNs expressing the same receptor converge onto one specific glomerulus [40]. Because transgenic *D. erecta* are still not available, we performed these experiments with *D. melanogaster* females expressing the Ca^2+^ sensitive reporter *GCaMP3.0* [24] from the *Orco* promoter to get an indication of which glomeruli and, consequently, which ORs were activated by 3M2BA. Stimulation with diagnostic odours and comparison with the map of the fly AL [34,41] enabled us to identify activated glomeruli (figure 3*a*–*c*). 3M2BA activated three glomeruli, DM2, DM5 and DM6, of which DM2 showed the strongest response (figure 3*a*). In *D. melanogaster*, DM2 receives input from ab3A type neurons that express the olfactory receptor Or22a [42]. This neuron type detects fruity esters in *D. melanogaster* [36] as well as in the other species of the *melanogaster* species group, including *D. erecta*. The signals measured from the DM5 and DM6 glomeruli probably stem from light scattering from the strongly activated neighbouring DM2 glomerulus.

To verify that ab3A OSNs in *D. erecta* underlie the responsiveness towards 3M2BA, we next performed SSR from the three large basiconic sensilla in this species. Indeed, these OSNs respond to 3M2BA in *D. erecta* (figure 3*d*). In addition, we also noted responses from the ab2A neuron towards 3M2BA, albeit only at high concentrations. Dose–response experiments performed from OSNs in ab2 and ab3 sensilla also demonstrate the high sensitivity of ab3A neurons to 3M2BA (figure 3*e*). We note that in the functional imaging experiments, no significant increased activity was observed from the DM4. This discrepancy is probably owing to the low sensitivity of the ab2A OSNs towards this substance, making it difficult to observe the signal with conventional functional imaging. In line with the notion that the signals from the DM5 stem from light scattering from DM2, we also recorded no increased spike firing in response to stimulation with 3M2BA from the ab2B OSNs, whose axons target DM5.

Interestingly, the specialization of *D. erecta* towards *Pandanus* thus appears to involve the same pathway as affected in *D. sechellia*. In *D. erecta*, the ab3A/DM2 pathway mediates information regarding the *Pandanus* volatile 3M2BA, whereas in *D. sechellia* the homologous pathway handles the major noni volatile methyl hexanoate. In *D. sechellia*, the increased sensitivity to the noni volatile is also accompanied by morphological alterations within the AL. Hence, we wondered if we would also find similar modifications in *D. erecta*.

### (d) Adaptation is mirrored in the internal and external olfactory organs

**Figure 4.**
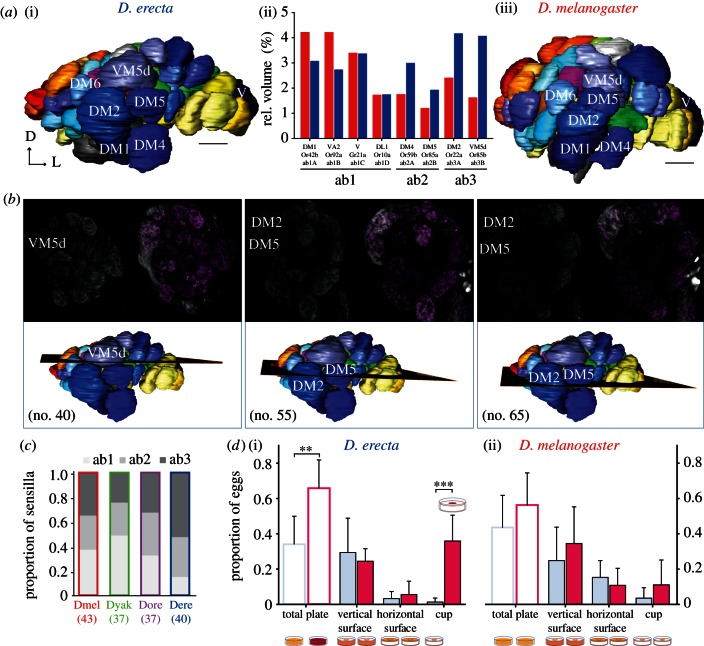
Morphological changes towards a complex of enlarged glomeruli. (*a*) Reconstruction of female antennal lobes (ALs) of the specialist *D. erecta* (i) and the generalist *D. melanogaster* (iii). Glomeruli terminology according to Couto [34]. ALs are viewed from medial to lateral. Comparison of the relative volume of the complex of enlarged glomeruli (based on the corresponding antennal input—large basiconic sensilla type ab1, ab2 and ab3) in *D. erecta* (blue), and *D. melanogaster* (red) (ii). The four glomeruli were up to 2.5 times enlarged in *D. erecta*, compared to the morphological structures in *D. melanogaster* (DM2×1.73; DM4×1.71; DM5×1.6; VM5d×2.5). *n* = 3 for *D. melanogaster*, *n* = 4 for *D. erecta*. Scale bar = 10 µm. Dorsal (D), lateral (L). (*b*) Neuronal backfill of ab3 sensilla in female *D. erecta*, viewed in three different planes of the AL. Labelled axons converge into the region of enlarged glomeruli (upper part). The corresponding planes are displayed in the reconstructed AL. Numbers in the paranthesis correspond to the plane (lower part). (*c*) Relative number of large basiconic sensilla of the four species under investigation [4]. Species name abbreviations according to the first three species letters. Total numbers of sensilla are given in parentheses. (*d*) Influence of 3M2BA in combination with spatial information (vertical structures) in oviposition site preference in the specialist *D. erecta* (i) and the generalist *D. melanogaster* (ii). Transparent bars represent relative number of eggs counted on the plates in total (control, light blue; 3M2BA, pink); solid bars include spatial information (vertical surface; horizontal surface; and vertical surface around odour cup). Mean ± s.d. Spatial preference of *D. erecta* and *D. melanogaster*: vertical medium surface > vertical gap around the odour cup > horizontal medium surface (*D. erecta*, *p* = 0.003; *D. melanogaster*, *p* = 0.002). 3M2BA significantly triggered oviposition in *D. erecta*, but not in *D. melanogaster* (*D. erecta*, *p* = 0.003; *D. melanogaster*, *p* > 0.05). Combination 3M2BA and spatial: *D. erecta* laid significantly more eggs inside the vertical gap around the odour cup > vertical medium structure (*D. erecta*, *p* < 0.001; *D. melanogaster*, *p* > 0.05). Per species, *n* = 6 cage of 30 flies.

To identify potential changes in the morphology of the olfactory system, we next reconstructed ALs of *D. erecta*, and compared these with the well-established AL structure of *D. melanogaster* (figure 4*a*). The AL of *D. erecta* revealed a complex of four enlarged glomeruli at the medial lateral site. Comparing these with the AL atlas of *D. melanogaster* [34,41,43], we identified these as homologous to DM2, DM4, DM5 and VM5d. The relative volume of all glomeruli was enlarged up to 2.5 times in *D. erecta*, compared with *D. melanogaster* (DM2 × 1.73; DM4 × 1.71; DM5 × 1.6; VM5d × 2.5; figure 4*a*(ii)).

The main factor contributing to the increase in glomerular volume is probably enhanced antennal input, i.e. a higher number of a certain sensilla [3]. In the *D. erecta* AL, the most enlarged glomeruli (DM2 and VM5d), presumably both receive input from OSNs housed in ab3 type sensilla. Indeed, using data from a previous study [4], we estimated the number of large basiconic sensilla for the species under investigation, and found a higher percentage of ab3 type sensilla in *D. erecta* compared with the other species (figure 4*c*). The increased volume of both DM4 and DM5, which receive input from OSNs housed in ab2 type sensilla can also be explained by a proportional increase of these sensilla in *D. erecta*. The larger spiking neuron in this sensillum type, which targets the DM4 glomerulus, also responded to 3M2BA, albeit only at high concentrations.

To further verify that the enlarged glomeruli in *D. erecta* receive input from ab3 type sensilla, we next labelled OSNs via anterograde-neurobiotin backfills, allowing us to connect physiological response with the neuronal correlates in the AL [3]. Indeed, the 3M2BA sensitive OSNs housed in ab3 type sensilla of *D. erecta* target the enlarged glomeruli (figure 4*b*).

In short, adaptations towards use of *Pandanus* in *D. erecta* parallel to a striking degree those found for the noni-specialist *D. sechellia*, even involving the same subpopulation of OSNs and receptor genes. However, some distinctions have to be emphasized here. First, in *D. erecta*, unlike *D. sechellia*, increased sensitivity is not based on the complete replacement of another type of sensilla (figure 4*c*). Second, while ab3A neurons in *D. erecta* are increased in numbers, the homologous neurons in *D. sechellia* also display a change in sensitivity (figure 3*e*).

### (e) 3-methyl-2-butenyl acetate triggers egg-laying in *Drosophila erecta*, but not in *Drosophila melanogaster*

Having unveiled the olfactory detection of host-derived signals in *D. erecta* females, we next examined the behavioural relevance of 3M2BA. Egg laying in *Drosophila* is a behaviour that is under strong selection pressure [44]. We, therefore, tested the specialist *D. erecta* and generalist *D. melanogaster* within a two-choice oviposition experiment by offering them either a control or 3M2BA (figure 4*d*). There are indications that flies preferentially lay eggs along vertical structures [45,46]. We, therefore, implemented spatial information, in addition to odour information, into our experimental set-up.

Considering the effect of spatial information, both *D. erecta* and *D. melanogaster* clearly preferred the vertical medium surface over the vertical gap around the odour cup, and over the horizontal medium surface (*D. erecta*, *p* = 0.003; *D. melanogaster*, *p* = 0.002; figure 4*d*; solid blue bars). However, 3M2BA only triggered oviposition in *D. erecta*, but not in *D. melanogaster* (*D. erecta*, *p* = 0.003; *D. melanogaster*, *p* > 0.05; figure 4*d*; transparent bars). More strikingly, this effect was strongly enhanced when combined with spatial information (vertical structures). In combination with 3M2BA, *D. erecta* laid significantly more eggs inside the vertical gap around the odour cup than along the vertical medium surface (*D. erecta*, *p* < 0.001; *D. melanogaster*, *p* > 0.05; figure 4*d*; solid pink bars).

Preferring vertical edges for egg-laying might be advantageous for flies in general, because it may provide protection from predators and desiccation. It is conceivable that both odour and spatial information are needed to indicate reliable egg-laying places. The potential role of 3M2BA as the sole stimulus triggering oviposition preference for *Pandanus* in *D. erecta* is unlikely. Most probably, it is a combination of *Pandanus* volatiles that guides gravid females to the fruits and induces egg-laying. However, an enhanced sensitivity towards a host odour with limited availability outside of the host, such as 3M2BA, would be evolutionary beneficial.

## 4. Conclusion

Finding an appropriate breeding and feeding site is a crucial aspect in an insect's life, because the success of that choice directly influences the offspring's nutritional intake and consequential fitness. In this regard, the sense of smell is critically important for many insects. Here, we studied the olfactory system of the specialist *D. erecta*, and provide evidence for adaptational changes in the insect towards its host and oviposition site, fruits of *Pandanus*. We not only found *D. erecta* being more sensitive towards host volatiles than its non-specialist siblings, we also demonstrate that the antennal response spectrum displays the life history rather than the phylogenetic relationship of the four flies investigated in this study. The higher sensitivity in *D. erecta* towards host volatiles is accompanied by changes in the AL morphology, specifically, a complex of enlarged glomeruli. We further uncovered the glomerular activation pattern and the receptor activation of 3M2BA, a novel *Drosophila* fruit ligand, which seems to play an important role in OSP of *D. erecta*.

In conclusion, olfactory adaptations in *D. erecta* appear to have occurred at two levels: first, at the periphery, with an increased number of ab3 sensilla; and second (as direct consequence), a shift towards a complex of enlarged glomeruli in the AL. One of these glomeruli, the DM2, is involved in processing the *Pandanus* key compound 3M2BA. Our results thus not only support previous findings in the noni fruit specialist *D. sechellia*, but also provide evidence for a general pattern of olfactory adaptations in insect–host associations.
